# Bipolar resistance switching characteristics with opposite polarity of Au/SrTiO_3_/Ti memory cells

**DOI:** 10.1186/1556-276X-6-599

**Published:** 2011-11-23

**Authors:** Xianwen Sun, Guoqiang Li, Li Chen, Zihong Shi, Weifeng Zhang

**Affiliations:** 1 Key Laboratory of Photovoltaic Materials of Henan Province and School of Physics and Electronics, Henan University, Kaifeng, 475004, People's Republic of China

**Keywords:** Au/SrTiO_3_/Ti, bipolar resistance switching, Schottky barrier

## Abstract

Two types of bipolar resistance switching with eightwise and counter eightwise polarities are observed to coexist in Au/SrTiO_3_/Ti memory cells. These two types of switching can be induced by different defect distributions which are activated by controlling the electric process. The analyses of *I-V *and *C-V *data reveal that the resistance switching with eightwise polarity originates from the change of Schottky barrier at the Au/SrTiO_3 _interface caused by trapping/detrapping effects at interface defect states, while the switching with counter eightwise polarity is caused by oxygen-vacancy migration.

## Introduction

Resistance switching between a high-resistance state [HRS] and a low-resistance state [LRS] by voltage pulses has recently attracted intensive attention for their potential application in the next-generation nonvolatile memory [1]. Many perovskite-type transition metal oxides, especially titanates [2-6], zirconates [7,8], and manganites [9-11], have been investigated as resistance switching materials. The resistance switching effect can be classified into two types: bipolar and unipolar [12,13]. Perovskite-type metal oxide devices generally exhibit bipolar resistance switching, in which the resistance state depends on the polarity of voltage. Two types of polarity behavior under the same bias voltage exist in the bipolar resistance switching. For the positive bias voltage, one is eightwise polarity, which changes resistance from a HRS to a LRS; the other is counter eightwise polarity, which converts a LRS into a HRS [14,15]. Up to now, the underlying mechanism for bipolar resistance switching is still a controvertible problem, and various models, such as Schottky-like barrier alteration [3,10], voltage-driven oxygen-vacancy migration [6], charge trapping in trap states [4], and so on, have been proposed to explain the switching behavior.

For bipolar resistance switching, an important issue is the physical origin of the switching polarity and their respective drive mechanism involved. Its clarification will be beneficial to get a comprehensive understanding of the switching mechanisms. These two switching types with eightwise and counter eightwise polarities occurring in the same medium have been discussed in the literatures [14-16]. We note that both kinds of polarity could be induced by choosing different top electrodes or modulating the range of applied voltage. These results actually imply the existence of different switching mechanisms in the same medium. Yang et al. reported that the redox reaction of the top electrode's oxide layer results in the bipolar switching with counter eightwise polarity, and the generation/annihilation process of the oxygen vacancy located at an oxygen-deficient layer at a metal/oxide interface contributes to the eightwise polarity [16]. Shibuya et al. showed that the conversion of switching polarity is due to the modification of the Schottky-like barrier and electron-trapping effect at the interface by applied fields [14]. Muenstermann et al. suggested allocating these two types of polarity to the filamentary or homogeneous conduction on the basis of conductive-tip atomic force microscopy topography [15]. Although these descriptions are not the same, they all recognize that the defect states are very important to the polarity conversion of these two switching types. The electric process can bring impacts about the defect density in the insulator/semiconductor [3,14]. The relationship between the resistance switching and the electric process is investigated in this paper in order to further understand the switching mechanism.

SrTiO_3 _[STO] films are considered as an n-type semiconductor due to the presence of oxygen vacancies [17]. In this work, we investigated the resistance switching performance of STO films which sandwiched between the Au top electrode and the Ti bottom electrode. The electric measurement results clearly show that the bipolar resistance switching originates from the Schottky junction formed at the Au/STO interface. Two types of bipolar switching with eightwise and counter eightwise polarities can be activated under different electric processes. The involved physical mechanisms and their relationship with defect states are investigated and discussed.

## Experimental details

**Figure 1:**
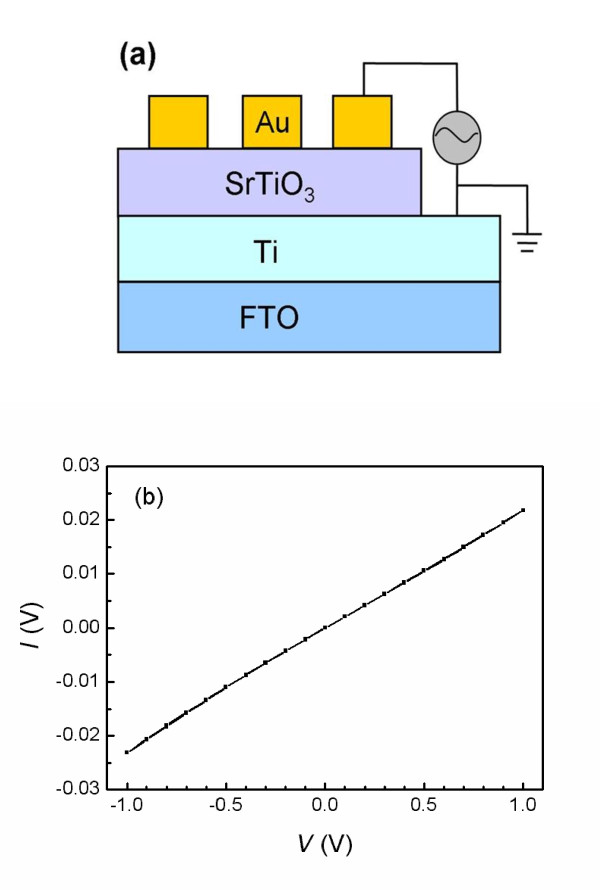
**The schematic diagram of the Au/STO/Ti cell and the *I-V *characteristics of the Ti/STO/Ti cell**. (**a**) The schematic diagram of the Au/STO/Ti cell and its measurement configuration. (**b**) The *I-V *characteristics of the Ti/STO/Ti cell. The linear curve shows an ohmic contact formed between the STO films and Ti electrodes.

To fabricate an Au/STO/Ti sandwich structure, a metal Ti film as the bottom electrode was deposited on a F-doped SnO_2 _conducting glass substrate by dc magnetron sputtering under an Ar pressure of 2.0 Pa. Then, a polycrystalline STO film was deposited on the Ti film by a pulsed laser deposition technique under a pressure of 2 × 10^-4 ^Pa at 600°C. Circular 100-nm-thick Au top electrodes with a diameter of 0.1 mm were sputtered onto the as-deposited STO films through a shadow mask by dc sputtering. Subsequently, the current-voltage [*I-V*] behaviors of the Au/STO/Ti cell were tested using a 2400 SourceMeter (Keithley Instruments, Cleveland, OH, USA). The capacitance-voltage [*Cp-V*] and parallel conduction-voltage [*Gp-V*] characteristics were investigated using a 4200 semiconductor characterization system (Keithley Instruments, Cleveland, OH, USA). We defined the current flowing from the Au top electrode to the Ti bottom electrode as positive. Figure 1a illustrates the structure of the Au/STO/Ti cell and its measurement configuration. To demonstrate ohmic contacts formed between the STO film and the low-work-function metal Ti, a Ti/STO/Ti cell was also prepared, and the *I-V *behaviors are shown in Figure 1b.

## Results and discussion

**Figure 2:**
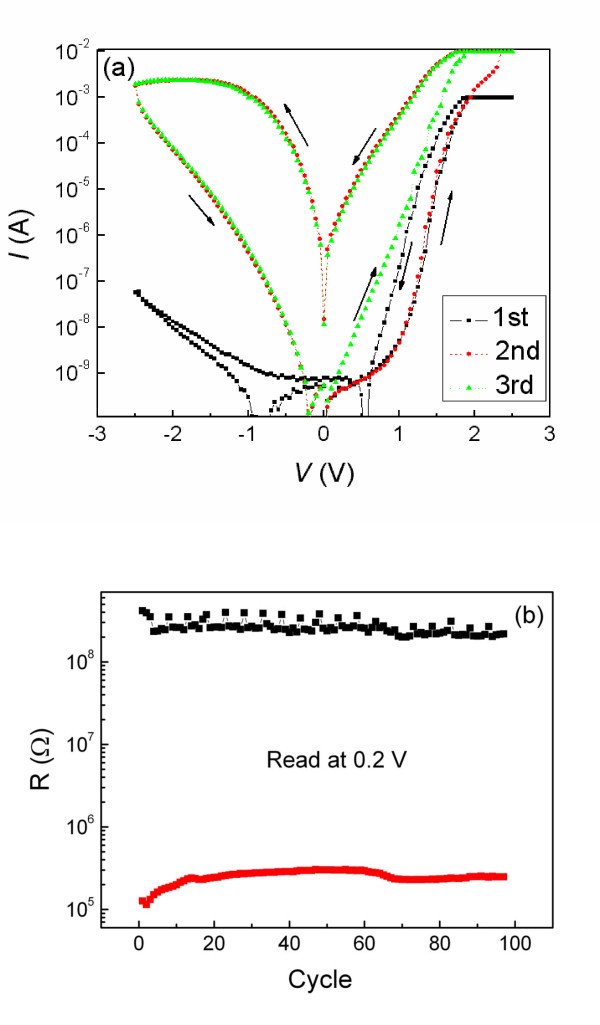
**Typical *I-V *characteristic of Au/STO/Ti cell and endurance property of bipolar switching with eightwise polarity**. (**a**) The typical *I-V *characteristic of the Au/STO/Ti cell for the first three cycles. The CC is 1 mA for the first cycle and 10 mA for the later cycles. (**b**) The endurance property of the bipolar switching with eightwise polarity. It can be repeated for 100 cycles without a difference.

Figure 2a shows the first three *I-V *cycles of the Au/STO/Ti cell for the voltage cycling of 0 → +2.5 → 0 → -2.5 → 0 V. A strong rectification behavior is observed, which exhibits an obvious and stable hysteresis with eightwise polarity after the second cycle. The switching from HRS to LRS occurs during the positive (forward) bias, and the reverse switching occurs during the negative (reverse) bias. Figure 2b demonstrates the endurance property of the Au/STO/Ti memory cell. It can be repeated for 100 cycles without a difference. The rectification behavior in the *I-V *is a typical feature of the Schottky junction, while the *I-V *hysteresis is a signature of nonvolatile resistance changes. The Ti/STO/Ti cell exhibits linear *I-V *curves, as shown in Figure 1b, which demonstrates that the ohmic contact is formed between the STO films and Ti electrode. Therefore, the rectifying *I-V *hysteresis of the Au/STO/Ti cell could be attributed to the presence of the depletion layer at the Schottky-like Au/STO interface. In addition, it is noteworthy that a shift of the current minimum from zero voltage exists in the forward and reverse *I-V *curves for the first cycle with 1-mA compliance current [CC], whereas the shift to positive from zero voltage vanishes, and the shift to negative still exists after the second cycle with 10-mA CC. This change of shift between the first and second cycles is relevant to the change of resistance state. When the cell is in a HRS, the positive or negative sweep voltages lead to a shift to positive or negative from zero voltage, respectively, whereas the shift from zero voltage vanishes when the cell is in a LRS, as shown in the positive branch of the second cycle and later cycles (Figure 2a). The Au/STO/Ti sandwich cell can be considered as a metal/oxide/metal capacitor, so the above phenomenon could be interpreted by the charging/discharging of the capacitor. The cell in HRS as a capacitor is charged into the opposite charges during the positive and negative sweep voltages, and the shift of current minimum from zero voltage represents the discharging process. However, the charges cannot be retained in the cell when it is in LRS, which demonstrates the existence of a large leakage current, so the shift of the current minimum from zero voltage vanishes.

**Figure 3:**
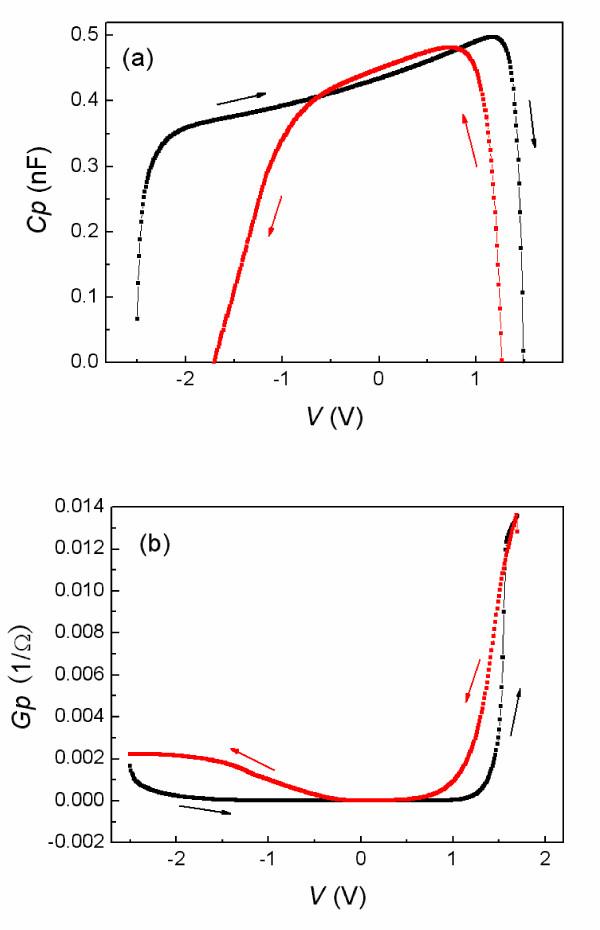
**The *Cp-V *and *Gp-V *characteristics**. (**a**) The *Cp-V *and (**b**) *Gp-V *characteristics were measured at 100 kHz with a test signal of 30 mV. The black and red curves are corresponding to the HRS and the LRS of the Au/STO/Ti cell, respectively.

To get more insights into the correlation between resistance switching and Schottky barrier, the *Cp-V *and *Gp-V *measurements were carried out at 100 kHz with a test signal of 30 mV. The measurement results are shown in Figure 3a, b. The arrows indicate the direction of the sweep voltage. The black and red curves match the HRS and LRS of the Au/STO/Ti cell, respectively. The capacitance decreases rapidly as the applied voltage increased, and approaches '0' under the voltage of about 1.5 and -2 V. Compared to the *Gp-V *curves in Figure 3b, the sharp decrease of capacitance corresponds to the increase of parallel conductance with the increased applied bias, which means that the sharp decrease of the capacitance is caused by the large leakage current under a higher voltage. While the change of capacitance is asymmetric in the positive and negative bias branches and there is a large variation in HRS and LRS, this assures a barrier height alteration which can be caused by charge trapping in interface states and/or oxygen-vacancy migration [18].

The above analysis shows that the bipolar resistance switching with eightwise polarity originates from the presence of the Schottky barrier near the Au/STO interface, in which there exist trapping states, such as oxygen vacancies and impurity states. The Schottky barrier width/height is changed due to the trapping/detrapping effect in the depletion layer [10,14]. When a large forward-bias voltage is applied to the Schottky junction, electrons are discharged from the trapping states, resulting in unoccupied trapping states in the Schottky barrier. The increased density of positively charged trapping states in the depletion layer reduces the Schottky width/height, which results in the large leakage current simultaneously. So the Au/STO/Ti cell is switched to the LRS. In the lower-bias-voltage region, electrons can tunnel easily through the reduced Schottky barrier. When a large reverse-bias voltage is applied, electrons are captured in the trapping states, resulting in the reduction of the net positive charge in the depletion layer. This makes the barrier wider and higher, and the cell returns to the HRS. The trapped electrons seem to be released during a coming positive bias sweeping. The electroforming process is not required to realize this eightwise-polarity switching. This is because the high concentration of defects exists in the pristine junction due to the STO films prepared in an anoxic environment.

**Figure 4:**
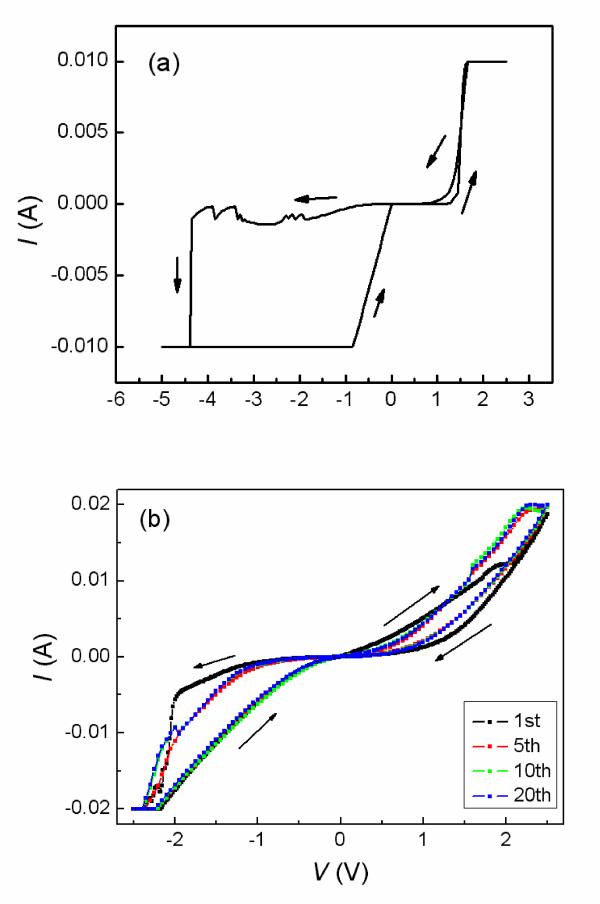
**Current transition process in negative bias and the bipolar resistance switching with counter eightwise polarity**. (**a**) The current transition process in the negative bias. (**b**) The bipolar resistance switching with counter eightwise polarity.

The Au/STO/Ti memory cell experiences a current transition when the negative bias increases to about -4.5 V. Then, the rectification behavior disappears, and nearly symmetric linear *I-V *curves were seen, as shown in Figure 4a. The interesting finding is that the bipolar resistance switching with counter eightwise polarity can be obtained when the sweep voltage is in the sequence of 0 → +2.5 → 0 → -2.5 → 0 V with 20-mA CC, as shown in Figure 4b. We can observe that the cell can only be switched firstly from the LRS to the HRS in the positive-voltage region. This switching type can be explained by the oxygen-vacancy migration, judging from the resistance change from LRS to HRS in the positive (forward) bias, as described for Sr_2_TiO_4 _films [14]. The oxygen vacancies, which are positively charged, are accumulated (extracted) into (from) the top interface region upon negative (positive) bias. In the process of the current transition, the Schottky-like barrier is considered to collapse due to the high density of oxygen vacancies concentrated at the Au/STO interface under negative bias. Simultaneously, the high density of defects are formed and extended within the active thin film, judging from its good conductivity and linear *I-V *behavior. So the oxygen-vacancy migration will be enhanced along extended defects [19]. The oxidization (reduction) of the interface defect states, which is caused by the oxygen vacancies migrating from (into) the top interface under positive (negative) bias, results in bipolar switching with counter eightwise polarity.

As stated above, the bipolar resistance switching with eightwise polarity originates from the change in the Schottky-like barrier height and/or width by trapping/detrapping effects at interface defect states, and the counter eightwise-polarity switching is caused by oxygen-vacancy migration. The current transition in Figure 4a is needed to convert the eightwise polarity to counter eightwise polarity. These findings can be explained as follows: In the initial state of the Au/STO/Ti cell, there exist trapping states in the Schottky junction. A strong electric field is applied to the depletion layer because the Schottky width is very small, so the trapping/detrapping effect of the defect states, which results in the change of barrier height/width, occurs at the depletion layer. Hence, the switching with eightwise polarity is realized. After the current transition, the Schottky barrier collapses, and the extended defects within the active thin film work as fast migration paths for oxygen vacancies, so the oxygen-vacancy migration is significantly enhanced along the extended defects. Thereafter, the cell exhibits counter eightwise-polarity switching based on an oxygen-vacancy-migration-related switching mechanism. In short, the switching mechanism is dominated by the defect state density of the active film, and the defect states can be controlled by controlling the electric process.

## Conclusions

In summary, we have investigated bipolar resistance switching characteristics of the Au/STO/Ti cell treated with different electric processes. The experiment results demonstrate that two types of bipolar resistance switching coexist in the same cell. The switching with eightwise polarity originates from the change in the Schottky-like barrier height and/or width by trapping/detrapping effects at the interface defect states, and the switching with counter eightwise polarity originates from oxygen-vacancy migration. The conversion from the eightwise polarity to the counter eightwise polarity is caused by the different defect distributions in the films which can be changed by different electric processes.

## Competing interests

The authors declare that they have no competing interests.

## Authors' contributions

XS carried out the experimental measurement and analysis and drafted the manuscript. GL participated in the experimental analysis. LC carried out the growth of Ti films. ZS participated in the growth of strontium titanate films. WZ carried out the experimental design. All authors read and approved the final manuscript.
